# 3 From Bottlenecks to Better Practices: Systemwide Electronic Clinical Decision Support for Blood Culture Stewardship

**DOI:** 10.1017/ash.2026.10460

**Published:** 2026-06-23

**Authors:** Darryl Nevels, Glodi Mutamba, Callyn Wren, Carolyn Stover, Christopher Evans, Becky Meyer

**Affiliations:** 1 Independent Contractor; 2 Tennessee Department of Health; 3 TN Department of Health

## Abstract

**Background:** Antibiotic prescribing varies across communities, and socioeconomic context may influence prescribing patterns. Prior studies have largely relied on aggregate deprivation indices, with limited integration of detailed neighborhood socioeconomic measures and prescriber-level Medicare Part D data. This study examined associations between census block-group socioeconomic characteristics, area deprivation, and antibiotic prescribing rates among Medicare Part D prescribers in Tennessee. **Methods:** Publicly available Medicare Part D (2023) antibiotic prescriber data were geocoded to exact practice addresses (n = 14,044) and linked to census block-group–level Area Deprivation Index (ADI) scores and socioeconomic indicators derived from the American Community Survey (ACS) 2019–2023 5-year estimates. Predictor variables included ADI decile and block-group measures of education, income, disability, veteran status, Social Security income, vehicle access, and health insurance coverage. Model diagnostics indicated over-dispersion; therefore, negative binomial regression with the logarithm of total claims as an offset was used to model antibiotic claims per prescriber. Incidence rate ratios (IRRs) and 95% confidence intervals (CIs) quantified associations. Additional models examined block-group–linked Medicare beneficiary race, ethnicity, and gender composition. **Results:** Higher ADI scores were modestly associated with increased prescribing (IRR = 1.01, p = 0.03), although this association was attenuated after accounting for rural–urban classification. Income-related indicators demonstrated the strongest relationships: block groups with a higher proportion of low-income residents exhibited substantially increased prescribing rates (IRRs ≈ 1.48–1.62, p < 0.001). Communities with greater reliance on Social Security income also had elevated prescribing (IRR ≈ 1.05, p < 0.001), as did disability prevalence (IRR = 1.06, p < 0.001) and limited vehicle access (IRR = 1.09, p < 0.001). Educational attainment and health insurance coverage were not consistently associated with prescribing patterns. In demographic models, block groups with higher proportions of Black (IRR = 0.92, p < 0.001) and Hispanic (IRR = 0.88, p < 0.01) Medicare beneficiaries were associated with lower prescribing rates, whereas higher proportions of male beneficiaries were associated with increased prescribing (IRR = 1.12, p < 0.001). **Conclusion:** Community socioeconomic context at the census block-group level was strongly associated with antibiotic prescribing among Medicare Part D prescribers in Tennessee. Income-related factors, disability prevalence, and limited mobility demonstrated the most consistent associations with higher prescribing rates. These findings underscore the importance of incorporating community context into antimicrobial stewardship strategies and support targeted interventions in areas where socioeconomic conditions may influence prescribing behaviors.

